# The relationship between attribution of blame and the perception of resistance in relation to victims of sexual violence

**DOI:** 10.3389/fpsyg.2022.868793

**Published:** 2022-08-25

**Authors:** Jesús de la Torre Laso, Juan M. Rodríguez-Díaz

**Affiliations:** ^1^Department of Social Psychology and Antropology, University of Salamanca, Salamanca, Spain; ^2^Department of Statistics, Faculty of Science, University of Salamanca, Salamanca, Spain; ^3^Institute of Fundamental Physics and Mathematics (IUFFyM), Faculty of Science, University of Salamanca, Salamanca, Spain

**Keywords:** attribution of blame, resistance, rape, group sexual offending, scenarios, victims, sexual violence

## Abstract

Several studies have examined victim blaming in rape scenarios. However, there is limited research on the analysis of the perception of blame when two or more perpetrators are involved. The present article explores the perception of blame in cases involving rape based on the level of resistance shown by the victim and the presence of one or more perpetrators. A study was carried out involving 351 university students who responded to a survey after reading a hypothetical assault scenario. Six situations were established where the victim showed either low or high resistance, depending on whether the resistance was verbal or physical and verbal, and in the presence of one or two male perpetrators. It is expected that perpetrators are more culpable when acting in groups and that less resistance from the victim leads to greater attribution of blame. The results confirm that more blame is attributed to the perpetrators when they act in groups than when they act alone. Likewise, women consider the victim generally exerts greater resistance and this variable influences the attribution of greater blame.

## Introduction

Sexual violence affects 100 of 1,000 of women every year. In Spain it is estimated that 8.9% of women have suffered sexual violence at some point in their lives by their current partner and 6.5% by someone with whom they do not have or have not had a relationship (Ministerio de Igualdad, 2019). In other countries, data show higher figures, as for example in the United Kingdom, where it is estimated that 20% of women have suffered a sexual assault (Wignall et al., 2020). However, a very small proportion of these situations are reported or end up in legal proceedings, as most of the episodes of sexual violence go unreported. This lack of reporting has given rise to the so-called black figure of crime (Datta and Bales, 2013). Among the circumstances in which victims do not report a sexual crime is the shame they experience in acknowledging they having been a victim; their state of confusion, guilt or shock after the assault; the fear of the offender and the perceived consequences of filing a complaint; the lack of witnesses or the difficulty in identifying the aggressors; the fear of not being believed; and distrust in institutions with regard to criminal proceedings (Drury et al., 2020). Another factor that discourages victims from reporting is the fear of being blamed for the crime, therefore, avoiding revictimization (Gutiérrez de Piñeres Botero et al., 2009).

The crime of sexual assault committed in a group is little known, although it is an internationally experienced reality. Studies have suggested that between 10% and 33% of sexual assaults have been committed by multiple perpetrators (Franklin, 2004). In South Africa, the country with the highest rates of multiple sexual assaults in the world, it would account for between one-third and one-half of all reported rapes (Artz and Kunisaki, 2003). In the United States, referrals range from 2% to 26% (Horvath and Kelly, 2009), in the United Kingdom they account for ~11%–19% of all sexual assault cases (Harkins and Dixon, 2013) and in South Korea it is estimated that 7.7% of sexual assault crimes involve two or more offenders (Park and Kim, 2016).

Studies on sexual violence have identified the importance of studying victims’ attribution of blame in order to determine the labels associated with women who have been sexually assaulted (Setia et al., 2020) and to prevent and avoid these situations (Rogers et al., 2009; Lim, 2017; Adolfsson et al., 2020).

In general terms, attribution of blame is understood as the preconception of blame and responsibility that is placed on a person in a negative event (Shaver, 1985). Frequently the victim is blamed in sexual crimes (Gerber et al., 2004; Randall, 2010). Attribution of blame is sometimes modulated by personal factors, such as the gender of the person assessing blame, also the gender of the perpetrator, his or her relationship with the victim, as well as situational factors such as rape myth acceptance of the person assessing blame, substance use, or the level of victim resistance (Grubb and Turner, 2012; Ayala et al., 2018).

It has been found that men assessing blame tend to blame the victim to a greater extent than women (Grubb and Harrower, 2009), which is explained because women tend to have higher levels of empathy towards rape victims and therefore tend to attribute greater credibility (Jimenez and Abreu, 2003). There are gender differences in the interpretation about certain relationship cues (such as having eye contact with another person, touching, flirting or going home with someone), and as Jozkowski et al. (2018) assert, men perceive these cues as consent to sexual activity, whereas women tend to see them as indicators of sexual interest, but not consent.

When it has been analyzed whether sexual offences are committed by acquaintances or strangers, research presents contradictory results (Grubb and Harrower, 2009); although rape victims who know their attacker are blamed to a greater extent than victims of stranger rape (Strömwall et al., 2013; Persson and Dhingra, 2020).

Another of the basic concepts associated with attribution of blame has to do with rape myths, first explained by Burt (1980). This concept captures those persistent and pervasive beliefs and attitudes that serve to exonerate the perpetrator and blame the victim for the rape. Four categories have been established: blaming the victim; excusing the perpetrator; beliefs that rape is not very serious; and that only certain types of women are raped (Gerger et al., 2013). Greater acceptance of rape myth is related to the crime being perceived as being less serious and greater victim blaming (Grubb and Turner, 2012). Some researchers have found that men are more likely than women to accept rape myths, and attribute greater victim blaming (Strömwall et al., 2014; Russell and Hand, 2017) although others have found a lack of differences between men and women (e.g., Abrams et al., 2003).

Substance use is also an influential element in the attribution of blame for an episode of sexual violence for both victims and perpetrators (Horvath, 2006); and in general, victims who consume alcohol are judged more harshly and are perceived by observers as more responsible for the attack than women who do not consume alcohol (Richardson and Campbell, 1982; Alicke, 2000).

However, regardless of any other factors, most people tend to attribute greater blame for the incident towards the perpetrator rather than the victim (Strömwall et al., 2013; Persson and Dhingra, 2020), and place minimal blame on the victim.

Victim resistance can affect the victim’s perception of blame for the sexual offence. Victim resistance relates to expressions of consent or refusal of sexual activity and, in particular, the form of verbal or non-verbal expression (Wignall et al., 2020). The absence of resistance has sometimes been associated with consent, but other studies have shown that this behavior, in the form of tonic immobility, is an involuntary and temporary state of motor inhibition, characteristic of sexual violence (Möller et al., 2017).

Previous studies have shown that in the case of verbal and physical resistance, is more likely to be considered rape when the woman only resists verbally (Shotland and Goodstein, 1983; Black and McCloskey, 2013). Likewise, an expression of physical resistance has been associated with a lack of consent in relationships (Ullman, 2007a) and, therefore, the absence of resistance may be perceived with increased victim blaming, and decreased perpetrator accountability (Davies et al., 2008).

This study aims to examine the circumstances under which victim blaming is attributed to rape. Victim blaming of rape has been shown to be associated not only with lower reporting rates, but also with a significant increase in the period required for psychological recovery (Ullman, 1996; Kline et al., 2021).

### Resistance, attribution of blame, and sexual offences committed by multiple offenders

In the last decade, the phenomenon of sexual offences jointly committed by more than two people or in groups has been analyzed (Morgan et al., 2012; Harkins and Dixon, 2013; among others). Attempts have been made to describe the circumstances of these offences (e.g., Horvath and Kelly, 2009; Quarshie et al., 2018), as well as the differences between assaults committed by a single perpetrator and by multiple perpetrators (Bijleveld and Hendriks, 2003; da Silva et al., 2015; Park and Kim, 2016). These studies focus on examining offender behavior (Chambers et al., 2010) or the impact of such crimes on the victims (Woodhams et al., 2007; Morgan et al., 2015).

In terms of the level of victim resistance, few studies have examined victim resistance in sexual assaults perpetrated by two or more people. However, those that do exist have found that victims of these crimes tend to show reduced levels of resistance (Porter and Alison, 2006), compared to assaults committed by a single subject (Wright and West, 1981; Hauffe and Porter, 2009). Wijkman et al. (2014) analyzed the cases of 25 women who had been sexually assaulted by groups of offenders and found that 31% of victims physically resisted the assault, while 12% expressed verbal resistance. According to Woodhams and Cooke (2013), depending on the level of violence shown by the perpetrators, the victim usually cognitively evaluates the situation and responds in such a way, increasing or decreasing resistance, in order to overcome or survive the assault. This psychological explanation serves to understand why when a woman is faced with a situation of danger to her physical and sexual integrity, she feels she must choose between survival and resistance. In the case of multiple offenders, the presence of more than one subject may deter victim resistance, reducing the need to display specific control strategies.

Most studies on attribution of blame in rape cases have typically used a scenario-based experimental method, where groups of observers read a hypothetical rape scenario with defined characteristics regarding the situation and the victim and then are asked to make judgments about these scenarios using quantitative rating scales (Sims et al., 2007; Adolfsson et al., 2020; Sjöberg and Sarwar, 2020). This approach is considered a good way to examine attribution of blame in rape offences because it minimizes the intrinsic difficulties of such studies and overcomes the associated practical, ethical, and sampling issues (Van der Bruggen and Grubb, 2014).

Studies on third party observer effects establish some relationship between the degree of victim resistance and victim blame. A recent study found that a victim’s attribution of blame in a hypothetical rape scenario increased for single-subject versus gang rapes, and that the blame increased as the degree of resistance decreased (Lim, 2017).

In contrast, researchers Adolfsson et al. (2020) examined the relationship between attribution of blame and the use of force in sexual assaults involving multiple perpetrators, and found higher levels of blame for victims of sexual crimes committed by a group of men compared to rape committed by a single perpetrator. Also, previous studies have previously detected greater negative social reactions to victims of sexual violence when the assault was carried out by a group of offenders (Ullman, 2007b).

On the other hand, there are few references that have analyzed the differences between the blame or responsibility of sexual offenders depending on whether they actively participate or remain as observers. According to Adolfsson et al. (2020), subjects who remain passive in a rape are also attributed a degree of blame, although this view may be conditioned by legislation and the punishment given to non-active participants.

The aim of this paper is to analyze the attribution of perceived blame in a hypothetical rape scenario, according to the different levels of resistance of the victim (verbal, physical, or both). In this study, in addition to the attribution of blame and resistance of the victim, the analysis of the role of a second aggressor who acts as an observer in the rape and does not carry out any sexual act with the victim was introduced. From a legal point of view, in Spain, the presence of a person or other persons who act in collusion with the one performing the forced sexual act is called a ‘necessary co-operator’. In these cases, the observer who is aware of the action being performed is also considered as if he or she were a direct perpetrator, even when there is no force involved, because it is considered that the intimidation exercised by such a figure weakens or annuls the victim’s will to be able to resist (Serrano, 2019).

Taking into account the objectives, the following hypotheses were put forward:

*H1*: Perpetrators are expected to be more perceived at fault when they act in groups.
*H2*: There will be less attribution of blame to the participant who does not actively participate in the sexual assault and only observes the sexual assault.
*H3*: Perceptions of victim resistance is expected to be lower in events involving more perpetrators.
*H4*: Less perceived resistance by the victim leads to higher attribution of blame.

## Materials and methods

### Design

**Figure 1:**
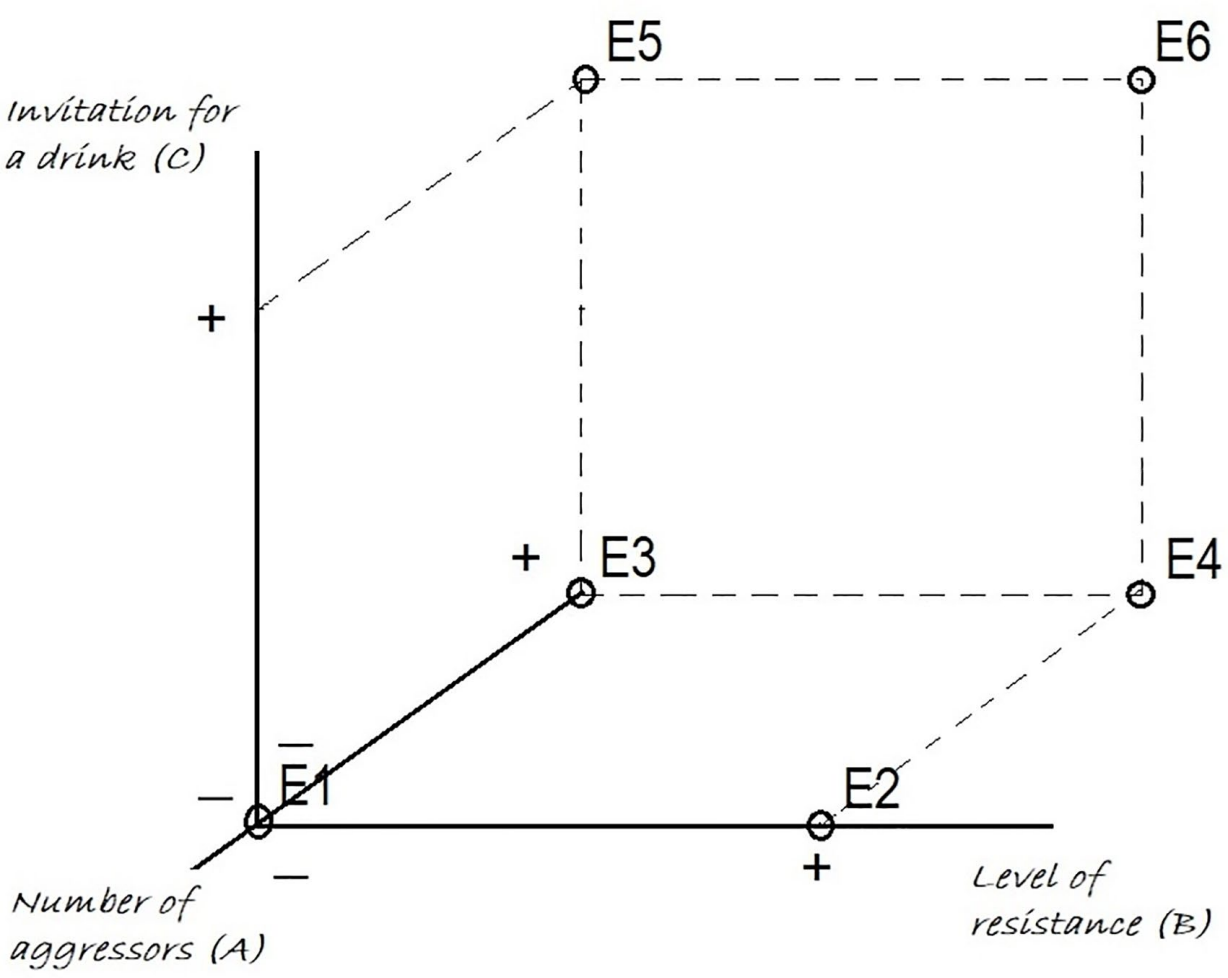
Scenarios.

Initially, a 2 × 2 design scenario was considered, using ‘Number of perpetrators’ (One-Two) and ‘Level of resistance’ (Low = verbal–High = physical) as independent factors. But eventually, the factor ‘origin of the invitation’ (Man–Woman) was added and crossed with ‘Level of resistance’ for the situations where there were two perpetrators, in order to assess whether the invitation of the woman affected the scenarios of sexual violence with several perpetrators. Thus six different scenarios were finally studied (Annex 1), with the following scheme (Figure 1):

### Participants

The study was conducted on a student population enrolled in the first 2 years of a bachelor’s degree in Criminology at the University of Salamanca. All the participants were Spanish. A total of 359 questionnaires were received from the participants, but eight were discarded because the verification questions had not been appropriately answered. Therefore, only the responses of 351 participants (290 women and 61 men) were taken into account. The average of the participants age was 20.9 years (*M* = 20.9; SD = 3.07; range 18–50).

### Materials

A total of six vignettes or scenarios were developed, similar to those used in other research studies (e.g., Rusinko et al., 2010; Lim, 2017). The scenarios in this study depicted a hypothetical situation of a date between a woman (Luna) and a man (Oliver) after meeting through an online dating application (Appex 1). The scenarios were manipulated based on the degree of resistance shown by the victim, and the number of perpetrators. This factor was dichotomized between high or low resistance. Low resistance was considered as the woman verbally refusing to have sex and high resistance when she expressed her refusal to have sex verbally and physically. Likewise, the word rape was not shown in the text so as to avoid a possible bias in subsequent classifications, in line with what has been established in other studies (Strömwall et al., 2014).

In the scenarios, the initiative to go home with someone, where the rest of the scene takes place, was differentiated between the man and the woman, although this condition was only taken into account in the scenarios in which there were two men. Other studies have shown scenarios involving duos or groups of three people (Adolfsson et al., 2020). However, since hardly any significant differences are observed when considering duos and larger groups, in this study, only the condition of two perpetrators has been presented as a group aggression.

### Questionnaire

To examine the attribution of blame, the questions used in previous studies (Krahé et al., 2008; Sleath and Bull, 2010; Lim, 2017) were adapted. As a result, a 12-item questionnaire was designed where the participant had to rate their level of agreement regarding each question based on a 7-point Likert scale (1 = none; 7 = totally). There were six questions related to the victim and six about the aggressor. For the questions indicating attribution of blame (for example: How much do you think Luna is to blame for what happened?) a high score reflected greater blame on the part of the victim and less attribution of blame on the part of the aggressor. The questions also addressed the explicit consent expressed by the victim (To what extent do you think Luna gave her consent?). The internal consistency of the 12 questions used was calculated with the 351 participants and is acceptable, with Cronbach’s alpha = 0.86.

A five-point scale (1 = no resistance, 5 = high resistance) was used to assess the difference between the victim’s level of resistance and the level of responsibility of both the victim and the aggressor. The last question examined the degree to which the scenario could be considered a rape (To what extent do you think a rape has taken place?).

Four questions were added to the questionnaires presenting a scenario involving two perpetrators (the second person with a passive role), and all questions involving the single aggressor (Oliver) were repeated to include the second aggressor (Dario), for the purpose of comparison.

#### Questionnaire verification

Two questionnaire verification questions were presented in this study. The first question asked about the number of men participating in the scenario and whether had been aware of what was going to occur. The second question asked whether the participant could identify with the individual who decided to go to the other person’s house. All questionnaires in which these two questions were not accurately answered were eliminated.

#### Demographic information

All participants were asked to indicate their age and gender. And at the end of the questionnaire, two questions were asked to determine whether the participant had ever been a victim of the scenario presented or knew someone who had experienced a similar situation.

### Procedure

The participants being invited to take part in a study on how people perceive and attribute responsibility based on an event involving a date between two young people who end up having sex.

The questionnaire was distributed to the participants *via* a form constructed using Google Forms. For its completion, participants were required to provide informed consent by ticking the option: ‘Yes, I agree to participate’ at the bottom of the first page before continuing with the questions. The participants were informed that the questionnaire was completely anonymous in order to control for the effects of social bias.

Participants were randomly assigned one of the six scenarios and the distribution per scenario was as follows: 53 participants in scenario 1; 62 in scenario 2, 60 in scenario 3, 60 in scenario 4, 60 in scenario 3 and 56 in scenario 3. Were asked to read the situation they had been presented with and answer the questions. The corresponding statistical analyses were carried out using the program spss v25.

The participants were randomly assigned to one of the six scenarios: 53 were assigned to scenario 1 (15.1% male and 84.9% female), 62 to scenario 2 (22.6% male and 77.4% female), 60 to scenario 3 (16.7% male and 83.3% female), 60 to scenario 4 (11.7% male and 88.3% female), 60 to scenario 5 (20% male and 80% female), 56 to scenario 6 (17.9% male and 82.1% female).

## Results

### Blame attribution

Table 1 shows the differences among the variables studied with regard to the level of blame attributed towards the victim. With respect to gender, Table 2 shows that men attribute more blame to the victim (*M* = 1.69; SD = 1.16) than women (*M* = 1.37; SD = 0.92).

**Table 1:** Different scenarios with variables.

Scenarios	A	B	C
E1	−	−	−
E2	−	+	−
E3	+	−	−
E4	+	+	−
E5	+	−	+
E6	+	+	+

A = Number of aggressors: One (−), Two (+); B = Level of resistance: Low (−), High (+); C = Invitation to go home for a drink: Man (−), Woman (+).

**Table 2:** Attribution of blame.

Variable	Levene’s test for equality of variances		*t*-Test for equality of means		
	*F*	Sig	*t*	df	Sig.
Participant’s gender	8.690	0.003^*^	1.997	77	0.049^*^
Number of perpetrators	5.23	0.023^*^	1.14	172	0.258
Participants who had been invited to go home with someone	0.648	0.422	0.286	0.234	0.775
Participants who had experienced a similar situation	4.60	0.033^*^	−1.475	103	0.143
Participants who knew someone who had experienced the same situation	6.73	0.010^*^	−1.889	287.47	0.60
Women participants who had experienced a similar situation	1.91	0.168	0.950	288	0.343
Women participants who knew someone who had experienced the same situation	3.459	0.064^**^	1.463	256	0.145

* *p* < 0.05;

** *p* < 0.1.

When analyzing the scenarios according to the number of perpetrators involved, the victim’s attribution of blame is greater in the scenarios where one perpetrator participate (*M* = 1.52; SD = 1.19) than those involving two perpetrators (*M* = 1.38; SD = 0.85). However, the difference is not significative, which does not allow us to confirm H1.

**Figure 2:**
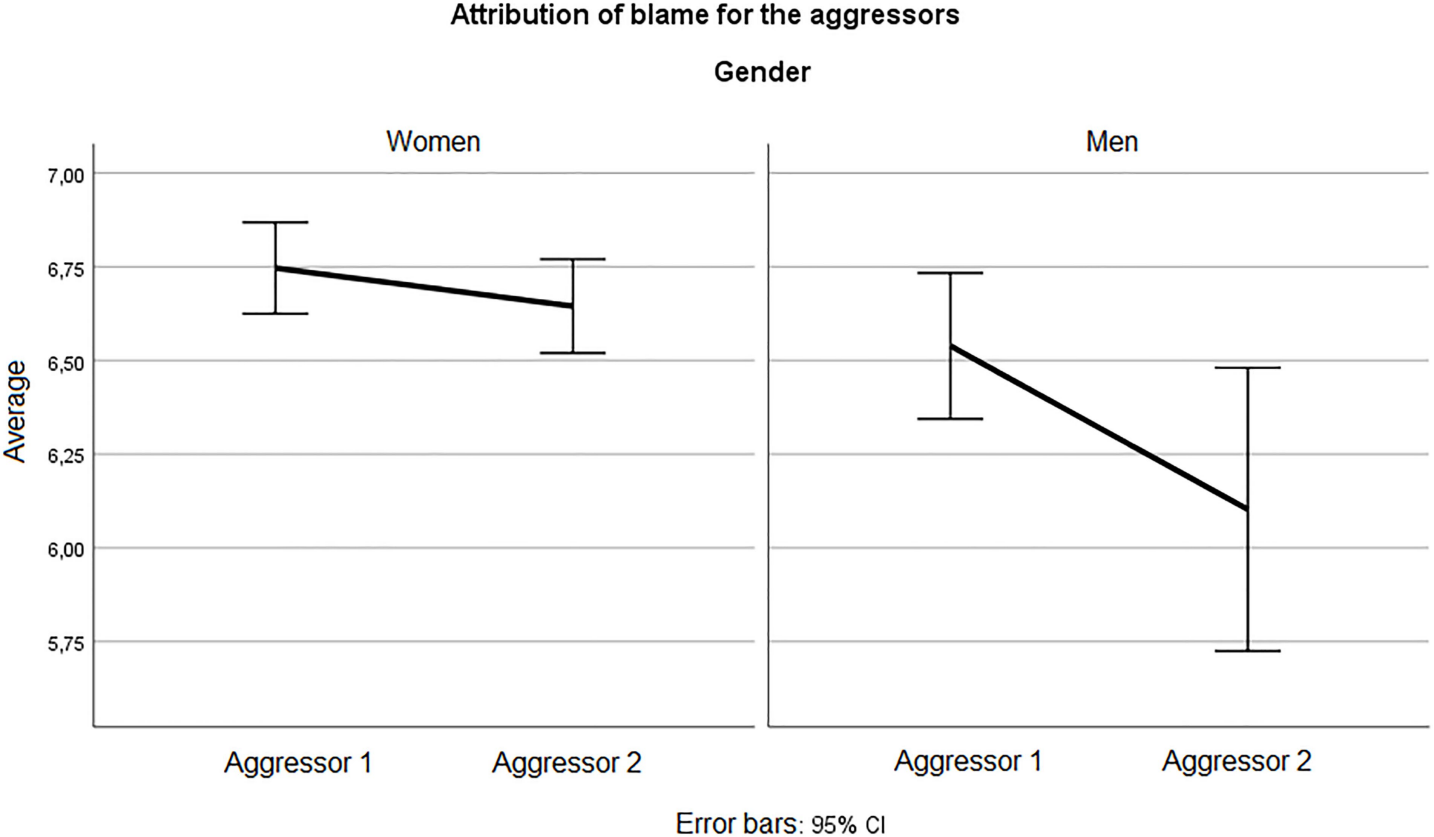
Attribution of blame according to gender.

Perpetrator 1 is considered the perpetrator in the one-man scenario and the one who had sex with the victim in the two-man scenario; perpetrator 2 is the one who acted as an observer in the scene of the two-man scenario. Overall, the results showed that attribution of blame was high for both perpetrators (Figure 2); however, perpetrator 2 was statistically attributed less blame than perpetrator 1 both for Men and Women (*t* (38) = 3.195, *p* = 0.003, and *t* (196) = 2.259, *p* = 0.025, respectively), confirming H2 hypothesis.

It can be seen that female participants attributed greater blame to the perpetrator who had sex with the woman (*M* = 6.75, SD = 0.87) versus the perpetrator who remained passive or perpetrator 2 (*M* = 6.64, SD = 0.89). In addition, female participants attributed significantly greater blame than males, (*t* (47.156) =2.751, *p* = 0.008), in the case of the perpetrator who does not have sex with the victim.

We were interested in determining whether the attribution of blame could vary according to whether one had experienced a similar situation directly or indirectly. A total of 65 participants had experienced a similar situation, but only three of them were men, thus only women were considered when studying this variable. The results show that the experience of direct victimization attributed less victim blaming (*M* = 1.27, SD =0.91) than those who did not have that direct experience (*M* = 1.40, SD = 0.92), although this difference was not significative (*p* = 0.343).

However, when considering the participants who knew someone else who had experienced a similar situation (165 participants, 87.27% of whom were female), the proportions by gender were similar to those of the participants in the study, and thus men were not removed for the study. In this case the results show a significative difference at the 10% significance level (*p* = 0.06) between the perception of blame of those who know someone who had gone through a similar situation (*M* = 1.33, SD = 0.91), compared to those who did not (*M* = 1.54, SD = 1.04).

### Resistance levels

Table 3 shows the victim’s levels of resistance observed according to the variables analyzed. The scenarios of low resistance (or verbal resistance) show lower levels than the scenarios of high resistance (physical and verbal resistance), confirming that this variable had been adequately measured. Thus, there were significant differences between the scenarios where the resistance was verbal (*M* = 3.34; SD = 0.95) compared to the scenarios where the victim expressed physical and verbal opposition (*M* = 4.41; SD =0 0.701).

**Table 3:** Resistance levels.

Variable	Levene’s test for equality of variances		*t*-Test for equality of means		
	*F*	Sig	*t*	df	Sig.
Low/high resistance	16.613	<0.001^*^	−12.100	349	<0.001^*^
Participant’s gender	4.654	0.032^*^	−2.341	81	0.022^*^
Number of perpetrators	0.205	0.651	0.891	349	0.373
Participants who had experienced a similar situation	0.386	0.535	−1.701	349	0.090^**^
Participants who knew someone who had experienced the same situation	1.732	0.189	1.723	308	0.086^**^
Women participants who had experienced a similar situation	0.000	0.994	2.158	288	0.032^*^
Women participants who knew someone who had experienced the same situation	4.859	0.028^*^	−1.863	230	0.064^**^

* *p <* 0.05;

** *p* < 0.1.

The participants’ perception of resistance was high, and women felt that the victim had shown more resistance than men *F* (4,654; 349) = −2.538, *p* < 0.05.

In general, women attribute higher levels of resistance towards the victim (*M* = 3.94, SD = 0.96) than men (*M* = 3.59, SD = 1.09). Conversely, no significant differences were observed between the scenarios involving 1 perpetrator (*M* = 3.95; SD = 0.99) or 2 (*M* = 3.85; SD = 0.99), thus H3 cannot be checked.

Upon analyzing whether the participants had had a similar experience, the score for attribution of resistance was lower (*M* = 3.69; SD = 0.92) than that for participants who had not experienced the same scenario (*M* = 3.92; SD = 1.00), *p* < 0.1. By contrast, victim resistance was scored higher (*M* = 3.94; SD =0.98) when the participant knew of someone who had experienced a similar situation than when they did not (*M* = 3.74; SD = 1.01), as well significative at the 10% significance level.

In a similar manner, we examined whether significant differences existed regarding the perception of victim resistance among women participants based on whether they had themselves experienced the same scenario or knew of someone who had. In these cases, the women who had first-hand experiences attributed a lower level of resistance to the victim (*M* = 3.71, SD = 0.91) than those who knew of someone who had had a similar experience (*M* = 4.01, SD = 0.91). However, less resistance was perceived among the women who had been directly victimized than those who had not personally experienced a similar situation, *p* < 0.05.

The fourth hypothesis, which proposed the relationship between attribution of blame and victim resistance, was assessed by means of a chi-square test. After dichotomizing both variables, the contrast was significant *χ*^2^ = 9.089, *p* = 0.002 thus supporting the relationship between these two factors.

Both male and female participants gave high scores with regard to whether the scenarios could be considered as rape (men: *M* = 6.25; women *M* = 6.57), with significative differences between them (*t* (349) = −2.289, *p* = 0.023). However, when this variable is analyzed in the context of the participant having suffered a similar situation or knowing someone who had, no significative differences were detected (*p* = 0.115 and *p* = 0.649, respectively).

## Discussion

The aim of this study was to examine the relationship between attribution of blame towards the perpetrators and towards the victim, within fictional rape scenarios, based on the level victim resistance, the degree of perpetrator involvement, and the origin of the invitation to go to a more private place.

In general, the data obtained support most of the hypotheses put forward. As expected, more blame is attributed to the perpetrators when they act in a group (in this case, as a duo), as opposed to when a man acts alone; thus, supporting H1 although the difference is not significative. This conclusion has been made previously in studies highlighting the importance of the presence of more than one perpetrator in attributing responsibility for sexual violence (Woodhams and Cooke, 2013; Lim, 2017; Adolfsson et al., 2020). Contrary to expectations, more blame was attributed to the perpetrator acting alone, as opposed to perpetrators acting in a group (in this case, as a duo), in line with Lim's (2017) research, but contrary to other similar research (Woodhams and Cooke, 2013; Adolfsson et al., 2020). Observers may perceive that responsibility is blurred in groups (Adolfsson and Strömwall, 2017).

Also, the perpetrators’ attribution of blame was much higher than that of the victim, as other research has found (e.g., Strömwall et al., 2014; Sjöberg and Sarwar, 2020). These results show that in rape cases the attribution of blame is polarized towards the perpetrators, thus emphasizing the importance of continuing such measurements in future research.

The individual characteristics of the participants (those filling in the questionnaire) explain much of blame attribution. For example, men tend to blame the victim to a greater extent than women, similar to observations found in other research (Alleyne et al., 2014). Likewise, in all the variables analyzed, there is greater variability in the opinions of men, which seems to indicate they do not have as clear an opinion as women. It is likely that differences in sex create gender bias whereby individuals identify with members of their in-group and have less favorable attitudes towards members of their out-group. In this case women are more likely to empathize more with the victims, which equates to less attribution of blame.

Furthermore, the fact that male participants attribute higher levels of blame to the victims and lower levels of blame to the perpetrators, compared to female participants, may have some practical implications for young people’s perceptions of rape victims and the acceptance of rape myths (Grubb and Turner, 2012), despite the fact that some authors consider that higher educational attainment results in a lower tendency to accept rape stereotypes (Idisis et al., 2007). Further studies could include examining whether other personal characteristics influence participants’ perceptions such as age, criminal history, or relationship to the victim, or whether socio-cultural factors may contribute to the assessment of blameworthiness, as Gravelin et al. (2019) argue.

As for the scenarios in which two perpetrators participate, the levels of blame are high for both. However, the participants (both men and women) attribute greater blame to the aggressor who has physical contact with the victim as opposed to the one who does not or remains in a passive role, confirming H2. As for this type of perpetrator, women tend to blame them more than men, as shown by the significant result. Another finding of this work is the high level of blame attributed to the perpetrator who does not act physically against the victim, which indicates that he/she is also perceived as a perpetrator. On the other hand, the lower level of blame perceived regarding the aggressor who does not exert physical contact with the victim may give a false sense of lower criminal responsibility. In Spain this aggressor could be considered as a perpetrator of the crime by being treated as an accomplice or “necessary co-operator” (Barrajón, 2019), or may be attributed a crime of omission of the duty to assist (Faraldo Cabana, 2020).

The direct or indirect experience of victimization was found to be influential in attributing less blame towards the victim. Those who had directly or indirectly experienced a similar situation attributed less blame to the victim than those who had not experienced it or did not know anyone who had been raped. It is plausible that direct victimization produces an empathy effect towards the victim and that indirect experience also influences perceptions, confirming the theory of the discrepancy that exists between the supposed “objective risk” of being a victim and the “subjective perceptions” of being a victim (Villalba Olivella, 2017).

As for the levels of resistance, although the scenarios showed levels far from low or high resistance, the average score of the victim in the scenarios selected as low resistance (3.34 on a scale of 5) were higher than expected. One explanation may be that the participants, mostly young people, are very aware of the rejection that a rape victim may experience and consider a verbal expression as a sign of lack of consent and, therefore, of resistance. Likewise, social movements such as #MeToo (Dio Bleichmar, 2018) or initiatives to change what is considered consent in sexual relationships (Bertolín-Guillén, 2021) may contribute to young people’s greater awareness of roles in these situations. Females attributed higher levels of resistance to the victim than males, confirming the different perceptions that males have of females in terms of signs or messages expressing explicit agreement or refusal in sexual relationships (Burkett and Hamilton, 2012; Jozkowski et al., 2018). Further research could confirm whether certain messages (e.g., accepting an invitation to go home with someone) are perceived by men and women differently. It could also be investigated whether the lack of resistance corresponds to the so-called tonic immobility (Gbahabo and Duma, 2021), which explains this lack of reaction in the victim.

No significative differences in the level of resistance were observed for scenarios in which one or two perpetrators acted, contrary to research that has reported that larger groups are associated with lower resistance (Woodhams and Cooke, 2013). The reason may be that the scenarios used for the groups are based on a small group of two perpetrators. Therefore, it would be necessary to confirm this relationship using larger perpetrator groups.

The hypothesis that predicted lower victim resistance leads to higher attribution of blame has been confirmed, a finding that is in line with previous research (Black and McCloskey, 2013; Lim, 2017). Therefore, it could be concluded that the victim’s level of resistance may influence the victim’s attribution of blame. In addition, direct experience of victimization is key to perceiving lower victim resistance, maybe feeling by own experience that the victim could have exerted more resistance than that shown in the scenery.

In the same way, the participants considered that the victim could not have avoided the incident, despite her resistance, a finding that confirms the belief in the inevitability of rape and how it contributes to the acceptance of rape myths (Burt, 1980). Likewise, considering that the victim could not have avoided the incident has consequences for the victims, as the severity of the emotional impact is related to the symptoms experienced (Sarasua et al., 2012; Echeburúa et al., 2013). Intervention through education and the elimination of victim stereotypes can contribute to preventing sexual violence. The links established between aggression against women and socially approved patterns of behavior, in terms of norms derived from male/female stereotypes, can help to understand the tolerance of sexual violence against women.

Although the scenarios did not include the word “rape,” the participants considered the events described as rape, especially among women who had experienced a similar situation. This outcome indicates that a non-consensual sexual experience is associated with an act of aggression.

## Limitations

There are a few limitations to this study. First, the study did not have a culturally diverse sample due to its location, and the applicability of these results is unclear. Cultural and regional differences are an important factor to be examined in future research to better generalize the results. This study was carried out on the basis of the questionnaires filled in by a sample of university students who are supposed to have a high cultural level. Therefore, more studies should be carried out using participants with different cultural levels, and over a wider age range. In addition, the younger population seems to be sensitized to the subject, owing to the fact that in recent years in Spain there have been different incidents of sexual aggressions committed in groups.

While attribution of blame and perception of resistance are complex concepts with a social component that are constructed according to the cultural roles within a given period, the results may lack variability. In this study, no distinction was made with regard to how blame is understood, and resistance was analyzed in terms of verbal and physical rejection. Thus, perhaps the distinction between low and high resistance has not been sufficient. Likewise, the simple account of blame attribution laid out in each scenario did not provide additional details that may have influenced how the event was perceived such as the absence of alcohol or the relationship between the aggressor and the victim. Consequently, providing a more complete description may have allowed other conclusions to be drawn.

## Conclusion

This study suggests that the blame attributed to victims and perpetrators in a rape scenario varies according to the gender of the participant filling in the questionnaire and the level of perpetrator involvement. Overall, participants attributed less blame to victims and more blame to perpetrators. Female participants attributed lower victim-blaming and higher perpetrator-blaming, regardless of their levels of involvement. However, men attributed different levels of blame between the perpetrators, with less blame being attributed to the passive perpetrator. On the other hand, the direct or indirect experience of having experienced a similar situation contributes to less blame being placed on the victim.

In addition, this study suggests that levels of resistance also vary according to the gender of the participant, with women perceiving the victim exerted more resistance.

The study has shown that there is an influence between levels of victim resistance and attribution of blame. Lower resistance is associated with greater blame. Currently, the literature is in line with these results, so it is necessary to design information and education strategies to have a better understanding of sexual assault.

## Data availability statement

The original contributions presented in the study are included in the article/Supplementary material, further inquiries can be directed to the corresponding author.

## Ethics statement

Ethical review and approval was not required for the study on human participants in accordance with the local legislation and institutional requirements. The patients/participants provided their written informed consent to participate in this study.

## Author contributions

JT contributed to developing the theoretical framework, data analysis, data collection, and the writing and overall writing of the manuscript. JR-D contributed to writing and data analysis of the manuscript. JT and JR-D contributed to editing and organization of the manuscript. All authors contributed to the article and approved the submitted version.

## Funding

This research was supported by the Spanish Ministry of Science and Innovation and Junta de Castilla y León (Projects, PID2021-125211OB-I00’ and ‘SA105P20’ respectively).

## Conflict of interest

The authors declare that the research was conducted in the absence of any commercial or financial relationships that could be construed as a potential conflict of interest.

## Publisher’s note

All claims expressed in this article are solely those of the authors and do not necessarily represent those of their affiliated organizations, or those of the publisher, the editors and the reviewers. Any product that may be evaluated in this article, or claim that may be made by its manufacturer, is not guaranteed or endorsed by the publisher.
