# Supplementary material for: The relationship between attribution of blame and the perception of resistance in relation to victims of sexual violence
Source: Front Psychol. 2022 Aug 25;13:868793. doi: 10.3389/fpsyg.2022.868793 (PMC9455198; doi:10.3389/fpsyg.2022.868793)
Supplement: Supplementary file 1 [file Data_Sheet_1.docx]

ANNEX 1: Scenarios

SCENARIO 1. Oliver and Luna met on a dating app, and, after exchanging a few messages, set up a date to get to know each other.

Oliver and Luna were happy and dressed to make a good impression. Oliver wore a shirt and a jacket, while Luna bought a new dress specifically for the date. They decided to go to a fast-food restaurant and after dinner, Oliver suggested to Luna to go back to his house for a drink; Luna accepted the offer.

Back at home, they talked a bit about their hobbies and interests, and during the conversation Oliver touched Luna's thigh. She smiled and move his hand away. After a bit more conversation, Oliver started to move closer to Luna and tried to kiss her. In order not to seem rude, she kissed him back. Oliver started touching her and suggested that they could have sex. Luna expressed that she did not want to have sex on the first date. Oliver and Luna continued to kiss and he began to remove her clothes. Luna mumbled again that she did not want to, but Oliver began to have sex with her.

SCENARIO 2. Oliver and Luna met on a dating app, and, after exchanging a few messages, set up a date to get to know each other.

Oliver and Luna were happy and dressed to make a good impression. Oliver wore a shirt and a jacket, while Luna bought a new dress specifically for the date. They decided to go to a fast-food restaurant and after dinner, Oliver suggested to Luna to go back to his house for a drink; Luna accepted the offer.

Back at home, they talked a bit about their hobbies and interests, and during the conversation Oliver touched Luna's thigh. She smiled and move his hand away. After a bit more conversation, Oliver started to move closer to Luna and tried to kiss her. In order not to seem rude, she kissed him back. Oliver started touching her and suggested that they could have sex. Luna expressed that she did not want to have sex on the first date. Oliver and Luna continued kissing, and he began to take off her clothes.

At this point, Luna pushed Oliver to try to get him off her, but she couldn't. Luna started screaming and kicking, but she couldn't get away. Oliver then started having sex with her.

SCENARIO 3. Oliver and Luna met on a dating app, and, after exchanging a few messages, set up a date to get to know each other.

Oliver and Luna were happy and dressed to make a good impression. Oliver wore a shirt and a jacket, while Luna bought a new dress specifically for the date. They decided to go to a fast-food restaurant, and after dinner Oliver suggested to Luna to go back to his house for a drink. On the way home they met Dario, a friend of Oliver's, who was then introduced to Luna.

During the conversation, Oliver asked Dario if he wanted to go back to his house and have a drink with them; Dario agreed.

While at Oliver’s house, they all talked a bit about their hobbies and interests, and during the conversation Oliver touched Luna's thigh. She smiled and moved his hand away. After a bit more conversation, Oliver started to move closer to Luna and tried to kiss her. In order not to seem rude, she kissed him back.

Oliver started touching her and suggested that they could all have sex. Luna expressed that she did not want to have sex on the first date. Oliver and Luna continued to kiss, and he began to take off her clothes.

Meanwhile, Dario stood in front of them, not allowing Luna to leave. Luna mumbled again that she did not want to, but Oliver began to have sex with her while Darius stood by watching.

SCENARIO 4. Oliver and Luna met on a dating app, and, after exchanging a few messages, set up a date to get to know each other.

Oliver and Luna were happy and dressed to make a good impression. Oliver wore a shirt and a jacket, while Luna bought a new dress specifically for the date. They decided to go to a fast-food restaurant, and after dinner Oliver suggested to Luna to go back to his house for a drink. On the way home they met Dario, a friend of Oliver's, who was then introduced to Luna.

During the conversation, Oliver asked Dario if he wanted to go back to his house and have a drink with them; Dario agreed.

Back at home, they all talked a bit about their hobbies and interests, and during the conversation Oliver touched Luna's thigh. She smiled and moved his hand away. After a bit more conversation, Oliver started to approach Luna and tried to kiss her. In order not to seem rude, she kissed him back.

Oliver started touching her and suggested that they could all have sex. Luna expressed that she did not want to have sex on the first date. Oliver and Luna continued to kiss, and he began to take off her clothes.

At this point, Luna pushed him to try to get him off her but was unsuccessful. Luna started screaming and kicking, but couldn't get away from him.

Meanwhile, Dario stood in front of them, not allowing Luna to leave. Oliver began to have sex with her while Dario stood by watching.

SCENARIO 5. Oliver and Luna met on a dating app, and, after exchanging a few messages, set up a date to get to know each other.

Oliver and Luna were happy and dressed to make a good impression. Oliver wore a shirt and a jacket, while Luna bought a new dress specifically for the date. They decided to go to a fast-food restaurant and after dinner, Luna suggested to Oliver to go back to her house to have a drink. On the way home they met Dario, a friend of Oliver's, who was then introduced to Luna.

During the conversation, Luna asked Dario if he wanted to go back to her house to have a drink with them; Dario agreed.

Back at home, they talked a bit about their hobbies and interests and during the conversation, Oliver touched Luna's thigh. She smiled and moved his hand away. After a bit more conversation, Oliver started to move closer to Luna and tried to kiss her. In order not to seem rude, she kissed him back.

Oliver started touching her and suggested that they could all have sex. Luna expressed that she did not want to have sex on the first date. Oliver and Luna continued to kiss, and he began to take off her clothes.

Meanwhile, Dario stood in front of them, not allowing Luna to leave. Luna mumbled again that she didn't want to, but Oliver began to have sex with her while Darius stood by watching.

SCENARIO 6. Oliver and Luna met on the dating app, and after exchanging a few messages, set up a date to get to know each other.

Oliver and Luna were happy and dressed to make a good impression. Oliver wore a shirt and a jacket, while Luna bought a new dress specifically for the date. They decided to go to a fast-food restaurant and after dinner, Luna suggested to Oliver to go back to her house for a drink. On the way home they met Dario, a friend of Oliver's, who was then introduced to Luna.

During the conversation, Luna asked Dario if he wanted to go back to her house to have a drink with them; Dario agreed.

Back at home, they talked a bit about their hobbies and interests, and during the conversation Oliver touched Luna's thigh. She smiled and move his hand away. After a bit more conversation, Oliver started to move closer to Luna and tried to kiss her. In order not to seem rude, she kissed him back.

Oliver started touching her and suggested that they all could have sex. Luna expressed that she did not want to have sex on the first date. Oliver and Luna continued kissing, and he began to take off her clothes.

At this point, Luna pushed him to try to get him off her but was unsuccessful. Luna started screaming and kicking, but couldn't get away from him.

Meanwhile, Dario stood in front of them, not allowing Luna to leave. Oliver began to have sex with her while Dario stood by watching.
